# Intimate partner violence during pregnancy and adverse birth outcomes: a case-control study

**DOI:** 10.1186/s12978-019-0670-4

**Published:** 2019-02-25

**Authors:** Eskedar Berhanie, Dawit Gebregziabher, Hagos Berihu, Azmera Gerezgiher, Genet Kidane

**Affiliations:** 1 Department of Nursing, College of Health Sciences and Comprehensive Specialized Hospital, Axum University, Tigray, Ethiopia; 2 Department of Anatomy, College of Health Sciences and Comprehensive Specialized Hospital, Axum University, Tigray, Ethiopia; 3 College of Health Sciences and Comprehensive Specialized Hospital, Axum University, Tigray, Ethiopia

**Keywords:** Intimate partner violence, Pregnancy, Birth outcome, Ethiopia

## Abstract

**Background:**

Intimate partner violence is a common phenomenon in Ethiopia families. About 81% of women believed that a husband is justified in beating his wife. About 30–60% of families were affected by their intimates. Women suffer physical, emotional, sexual and economic violence by their intimate partners. It often remains either for the sake of family secrecy, cultural norms or, due to fear, shame and community’s reluctance on domestic affair and social stigma.The objective of this study is to examine the association between intimate partner violence during pregnancy and adverse birth outcomes.

**Methods:**

A hospital based unmatched case control study was conducted in four zonal hospitals of Tigray region. A total of 954 study participants (318 cases and 636 controls) were taken. Systematic sampling was used to select the cases and controls. Ethical clearance was obtained throughout the study period.

**Result:**

Out of 954 interviewed mothers, 389 (40.8%) had experienced intimate partner violence during their index pregnancy period. More than two third (68.6%) of cases had been exposed to intimate partner violence. Multivariable logistic regression analysis showed that, women exposed to intimate partner violence during pregnancy were three times more likely to experience low birth weight (AOR = 3.1; CI 95% [1.470,6.618]) and preterm birth (AOR = 2.5; CI 95% [2.198–2.957]). It was observed that women who had been exposed to physical violence during pregnancy were five times more likely to experience low birth weight (AOR = 4.767; CI 95% [2.515, 9.034]) and preterm birth (AOR = 5.3; CI 95%: 3.95–7.094).

**Conclusion and recommendation:**

It was found that the risk of preterm birth and low birth weight was increased when the pregnant women were exposed to more than one type of intimate partner violence and physical violence during pregnancy. Therefore, Efforts to address maternal and newborn health need to include issues of violence against women.

**Electronic supplementary material:**

The online version of this article (10.1186/s12978-019-0670-4) contains supplementary material, which is available to authorized users.

## Plain English summary

Violence against women is recognized as a significant public health problem, with serious consequences for women’s physical, mental, sexual, and reproductive health. The most common type of violence against women is intimate partner violence, which refers to any behavior within an intimate relationship that causes physical, psychological or sexual harm to those in the relationship. Intimate partner violence during pregnancy that it may confer risks to the neonate through the mother’s increased risk of premature birth, as well as the infant being at risk for low birth weight.

This study focused on to examine the association between intimate partner violence during pregnancy and adverse birth outcomes.

Women who gave birth in the selected hospitals, during the study period were interviewed during the study period. Women’s who had been exposed to intimate partner violence during pregnancy were more likely to have low birth weight and preterm baby.

Face to face interview was done to a total of 954 women who participated in the study with a response rate of 100%. The mean (±SD) age of mothers was 28.37 ± 6.25 years’ ranges from 16 to 48 years. Seventy (22.0%) of cases and 138(21.7%) controls were living in rural areas. Regarding to marital status, 276(86.8%) cases and 554(87.1%) of controls were married. Two hundred nine (65.7%) of cases and 367(57.7%) of controls were house wives by occupation and 43(13.5%) cases and 85(13.4%) of controls had not attended formal education.

## Background

Violence is the intentional use of physical force or power, threatened or actual, against oneself, another person, or against a group or community, which either result in or has a high likelihood of resulting in injury, death, psychological harm maldevelopment or deprivation [1].

Women’s health and lives, in particular, are seriously affected by gender-based violence. Violence against women, committed by an intimate partner, is recognized as a significant public health problem and Human Rights issue. Violence by an intimate partner is manifested by physical, sexual or emotional abusive acts as well as controlling behaviors [2]. Though violence occurs in different forms and settings including workplace, school, and community, violence at home by intimate partner violence is considered as the most prevalent form [3].

According to UN declaration, violence against women includes “any act of gender-based violence that results in, or is likely to result in, physical, sexual or psychological harm or suffering to women, including threats of such acts, coercion or arbitrary deprivations of liberty, whether occurring in public or private life [4].

Global estimates published by WHO indicate that about 1 in 3 (35%) of women worldwide have experienced either physical and/or sexual intimate partner violence or non-partner sexual violence in their lifetime. Most of this violence is intimate partner violence. Worldwide, almost one third (30%) of women who have been in a relationship report that they have experienced some form of physical and/or sexual violence by their intimate partner in their lifetime [5]. Intimate partner violence is also widespread in Sub-Saharan Africa; surveys conducted in the region showed that 46% of Ugandan women, 60% of Tanzanian women, 42% of Kenyan women, and 40% of Zambian women reported regular physical abuse [6].

Intimate partner violence is a common phenomenon in Ethiopia both in urban and rural families. About 81% of women believed that a husband is justified in beating his wife. Surveys in Ethiopia have also reported that nearly one-half to two-thirds of ever-partnered women experienced intimate partner violence [7, 8]. Women suffer physical, emotional, sexual and economic violence by their intimate partners. It is often protected by family secrecy, cultural norms, fear, shame, community’s reluctance on the domestic affair and social stigma [9].

According to the Center for Disease Control of the United States, (CDC), pregnancy may represent a unique opportunity for the vulnerable woman to have contact with health care providers, making it an important time for the detection of violence during and after the pregnancy [10]. The prevalence rate of intimate partner violence during pregnancy ranging between 4 and 29% [11].

Intimate partner violence (IPV) during pregnancy does not only affect the women’s reproductive health, but also imposes fatal and non-fatal adverse health outcomes on the growing fetus due to the direct trauma of abuse to a pregnant woman’s body, as well as the physiological effects of stress from current or past abuse on fetal growth and development [12]. A substantial amount of research supports that IPV during pregnancy confers risks to the neonate by increasing premature birth (PTB) as well as the infant being at risk for low birth weight (LBW). LBW and PTB are well established leading causes of neonatal morbidity and mortality [13].

Given the magnitude and severity of the problem, there is a great need for the generation of evidence-based information to appropriately respond to the problem. Despite the abundance of many studies, the role of violence as an underlying factor in women’s health during pregnancy and birth outcome remains an area where robust evidence is lacking. To serve the intervention programs, there is a need to generate information on the association between IPV and adverse neonatal outcome. In doing so, this study was aimed to contribute information which would be filling the existing information gap. The policy environment in the area of women’s health and women’s right are current efforts of the government taken to address the problem. The study of this kind can help the policy makers to look in to the grassroots level whether the existing policy issues are well understood by the public at large and to make further adjustments whenever needed. Furthermore, it may also serve as a base line data in identifying potential research areas for further studies.

Nurses and other obstetric care providers working in maternal and child health centers as well as the community setting can use the finding of this study during prenatal education to inform couples about the risk of adverse birth outcomes in relation to intimate partner violence exposure.

## Methods

A hospital-based unmatched case control study design was conducted in Tigray Regional state of Northern Ethiopia, from March to January 2017 in four randomly selected Zonal Public Hospitals. Tigray forms the northernmost reaches of the nine ethnic regions of Ethiopia and is located between 36 ° and 40 °’ East longitude. Its North-South extent spans 12 and a half degrees to 15 ° north. It is bordered by Eritrea in the North, Sudan to the West, Amhara national state to the Southwest and Afar of Ethiopia to the East. The region covers 54,572 km^2^ ranging from low-arid to highland (above 2200 masl) areas. Tigray is subdivided into six administrative zones such as Central zone (Aksum), Eastern zone (Adigrat), North Western zone (shire), Southern zone (Michew), Western Zone (Humera), Mekelle (special zone). Tigray has an estimated population of 5,247,005 according to the 2009 EFY population estimation. Among those 2,660,002 are Females and 2,587,003 are Males [14].

The source and study populations for this study were all women who gave birth in selected Zonal Hospitals of Tigray, Ethiopia. All sampled women who gave birth in selected were also studied population for this study. Cases were all women who had adverse birth outcomes (neonates born prematurely and with low birth weight) and Controls were all women who had a normal birth outcome (neonates born at term and with normal birth weight) in the selected zonal hospitals of Tigray, Ethiopia, during the study period.

Data were collected by face to face interview method using a pre-tested structured questionnaire (Additional file 1). It consisted of sociodemographic, obstetric, and experiences-of-violence related questions. Four female nurses and supervisors were recruited as interviewers and as supervisors respectively. Data collectors and supervisors were trained for two days on techniques of interviewing, the purpose of the study, importance of privacy, sensitivity of the issue, discipline and approach to the interviewees and confidentiality of the respondents.

Adverse birth outcomes were measured by low birth weight, preterm birth. Low birth weight was defined as a live birth weighing < 2500 g. Preterm was defined as a neonate born before 37 completed weeks. The cut off points used for the birth outcomes were based on standards [17]. Intimate partner violence during pregnancy was assessed by asking women if she had experienced any act of physical, sexual or psychological abuse during index pregnancy by intimate partner. Controlling behaviors were defined as isolating a person from family and friends; monitoring their movements; and restricting access to financial resources, employment, education or medical care. Index pregnancy period, in this study refers to all trimesters of last pregnancy.

The data were checked for completeness and inconsistencies, then entered using Epi-Info version 7 and cleaned and analyzed in SPSS version 21. Cross-tabulation was done to see the distribution of cases and controls. Descriptive statistics were used to characterize the sample and numerical data and was presented as mean + SD, median ± interquartile range, proportion or percentages. The binary logistic regression model was employed to examine the relationship or statistical association between the outcome variable and selected independent variables. All variables with a *P* value < 0.05 were included in the multivariable analysis.

A multi-variable analysis was carried out to evaluate the association between intimate partner violence and adverse birth outcome after adjusting for confounding variables. Results were presented as adjusted odds ratios (AOR) with 95% CI, which express the magnitude of the effect of each category on the outcome relative to the reference category. The significance level was set at *P*-value (< 0.05). Results were presented using tables and texts.

Ethical clearance was obtained from the institutional review board (IRB) of Aksum University, College of Health Science. Official permission was obtained from the Tigray regional health bureau. In addition, the ethical considerations were done by considering the personal and revealing nature of the study, which needed the required voluntary and informed consent to be obtained from the participants. Prior to administering the questionnaires, the objectives of the study were clearly explained to the participants and verbal consent was obtained. Confidentiality and anonymity were ensured throughout the execution of the study.

## Result

### Socio-demographic characteristics of respondents

In this study, a total of 318 women’s who had the adverse birth outcomes (cases) and 636 women’s who had the normal birth outcome (controls) were included making a response rate of 100%. The mean (±SD) age of mothers was 28.37 ± 6.25 years’ ranging from 16 to 48 years. Seventy (22.0%) of cases and 138(21.7%) controls were living in rural areas. Regarding marital status, 276(86.8%) cases and 554(87.1%) of controls were married. 209 (65.7%) of cases and 367(57.7%) of controls were housewives by occupation and 43(13.5%) cases and 85(13.4%) of controls had not attended formal education. (Table 1 show in a separate document).

**Table 1:** Distribution of socio demographic characteristics of cases and controls who gave birth in public zonal hospitals of Tigray, Ethiopia, 2018

Variables	Category	Cases *n* = 318 (%)	Control *n* = 636 (%)	Total *n* = 954(%)
Age group	< 20	27 (8.5%)	72 (11.3%)	99 (10.4%)
	21–34	228 (71.7%)	422 (66.4%)	650 (68.1%)
	> 35	63 (19.8%)	142 (22.3%)	205 (21.5%)
Ethnicity	Tigray	294 (92.5%)	576 (90.6%)	870 (91.2%)
	Amhara	18 (5.7%)	32 (5.0%)	50 (5.2%)
	Afar	4 (3.8%)	22 (3.5%)	26 (2.7%)
	Others^a^	2 (0.6%)	6 (0.9%)	8 (0.8%)
Religion	Orthodox	272 (85.5%)	511 (80.3%)	783 (82.1%)
	Islam	14 (4.4%)	65 (10.2%)	79 (8.3%)
	Catholic	14 (4.4%)	38 (6.0%)	52 (5.5%)
	Protestant	18 (5.7%)	22 (3.5%)	40 (4.2%)
Marital status	Single	24 (7.5%)	49 (7.7%)	73 (7.7%)
	Married	276 (86.8%)	554 (87.1%)	830 (87%)
	Divorced / Widowed	18 (5.2%)	33 (5.2%)	51 (5.3%)
Educational level	No education	43 (13.5%)	85 (13.4%)	128 (13.4%)
	Primary school	70 (22.0%)	123 (19.3%)	193 (20.2%)
	Secondary school	111 (34.9%)	213 (33.5%)	324 (34.0%)
	Preparatory school	19 (6.0%)	31 (4.9%)	50 (5.2%)
	Collage/university	75 (23.6%)	184 (28.9%)	259 (27.1%)
Occupation	House wife	209 (65.7%)	367 (57.7%)	576 (60.4%)
	Employed	92 (28.9%)	259 (40.7%)	351 (36.8%)
	Student	17 (5.3%)	10 (1.6%)	27 (2.8%)
Monthly income	≤500 Birr	51 (17.9%)	112 (18.8%)	163 (18.5%)
	501–1000 Birr	150 (52.6%)	228 (38.3%)	378 (42.9%)
	1001–1500 Birr	17 (6.0%)	42 (7.0%)	59 (6.7%)
	> 1500 Birr	67 (23.5%)	214 (35.9%)	281 (31.9%)
Place of Residence	Urban	248 (78.0%)	498 (78.3%)	746 (78.2%)
	Rural	70 (22.0%)	138 (21.7%)	208 (21.8%)

^a^Others: Debub, Ethiopian Somalie, Eritrea, Oromia

### Pregnancy and obstetric characteristics of mothers

The median (±IQ range) of parity for both cases and controls was 2 ± 2 ranges from 1 to 10 live births. Majority of the respondents, 632(99.4%) controls and 311(97.8%) cases had received ANC service at least once during the index pregnancy. More than one thirds, 281(44.2%) of controls and 136(42.8%) cases had > 3 number of pregnancies. (Table 2 show in a separate document).

**Table 2:** Pregnancy and obstetric characteristics of mothers among cases and controls who gave birth in public zonal hospitals of Tigray, Ethiopia, 2018 (*n* = 954)

Variables	Category	Cases *n* = 318 (%)	Control *n* = 636 (%)	Total *n* = 954(%)
ANC follow up	No	7 (2.2%)	4 (0.6%)	11 (1.2%)
	Yes	311 (97.8%)	632 (99.4%)	943 (98.8%)
Number of ANC visit	1–3 times	82 (25.8%)	148 (23.3%)	230 (24.1%)
	> 4 times	236 (74.2%)	488 (76.7%)	724 (75.9%)
Parity	Primipara	134 (42.1%)	236 (37.1%)	370 (38.8)
	Multipara	184 (57.9%)	400 (62.9%)	584 (61.2%)
Gravida	1	120 (37.7%)	220 (34.6%)	340 (35.6%)
	2	62 (19.5%)	135 (21.2%)	197 (20.6%)
	> 3	136 (42.8%)	281 (44.2%)	417 (43.7)
Pregnancy Intention	Intended	302 (95.0%)	595 (93.6%)	897 (94.0%)
	Unintended	16 (5.0%)	41 (6.4%)	57 (6.0)

Unintended pregnancy: refers to pregnancies that are mistimed, unplanned or unwanted at the time of conception

Abbreviations: *ANC* antenatal care

### Types of intimate partner violence during pregnancy

More than half, (68.6%) cases and one fourth (26.9%) of controls reported experiencing some form of IPV during the index pregnancy, 183 (57.5%) cases and 51(8.0%) of controls reported experiencing some act of physical violence during their index pregnancy and one third of cases 103 (32.4%) and 26 (4.1%) of controls reported being slapped by their intimate partner as the most common type of physical violence during pregnancy.

Less than one fourth, (22.0%) of the cases and 75(11.8%) of the controls reported experiencing some act of sexual violence during pregnancy. Sixty-six (20.8%) of the cases reported having unwanted sexual intercourse, because they are scared of what their partner might do if they do not agree. This was the most common sexual violence followed by being forced to do something sexual that felt degrading or humiliating. 52(16.4%) and 38(11.9%) being Physically forced to have sexual intercourse.

Similarly, the proportion of controls reported to be having unwanted sexual intercourse for fear of violence by their partner was 70(11.0%) followed by being forced to do something sexual that felt degrading or humiliating 51(8.0%) and 31(4.9%) were physically forced to have sexual intercourse.

Those women who had been exposed to intimate partner violence during pregnancy period (68.6%) had a pre-term and LBW infant. Of these,135(42.5%) were as a result of exposure to physical violence. Some form of psychological violence was also experienced by 104(32.7%) cases and 115(18.1%) of controls. Insulting 104(32.7%) were the commonest form psychological or emotional violence reported during their index of pregnancy (Table 3 show in a separate document).

**Table 3:** Types of intimate partner violence’s during pregnancy among women who gave birth in public zonal hospitals of Tigray, Ethiopia, 2018 (*n* = 954)

Variables	Category	Cases *n* = 318 (%)	Control *n* = 636 (%)	Total *n* = 954(%)
Over all violence	No	100 (31.4%)	465 (73.1%)	565 (59.2%)
	Yes	218 (68.6%)	171 (26.9%)	389 (40.8%)
Physical violence (*n* = 156)				
Slapped	No	215 (67.6%)	610 (95.9%)	789 (82.7%)
	Yes	103 (32.4%)	26 (4.1%)	165 (17.3%)
Pushed/shoved/pulled your hair	No	215 (67.6%)	610 (95.9%)	825 (86.5%)
	Yes	103 (32.4%)	26 (4.1%)	129 (13.5%)
Kicked/dragged or beating	No	302 (95.0%)	626 (98.4%)	928 (97.3%)
	Yes	16 (5.0%)	10 (1.6%)	26 (2.7%)
Hit with fist/something else that could hurt you	No	311 (97.8%)	625 (98.3%)	936 (99.2%)
	Yes	7 (2.2%)	11 (1.7%)	18 (0.8%)
Choked or burnt you on purpose	No	315 (99.1%)	631 (99.2%)	946 (99.2%)
	Yes	3 (0.9%)	5 (0.8%)	8 (0.8%)
Threatened or used a weapon against you	No	314 (98.7%)	636 (100%)	950 (99.6%)
	Yes	4 (1.3%)	0 (0.0%)	4 (0.4%)
Psychological violence (*n* = 156)				
Insulted or made you feel bad about yourself	No	214 (67.3%)	521 (81.9%)	735 (77.0%)
	Yes	104 (32.7%)	115 (18.1%)	219 (21.3%)
Belittled or humiliated you in front of others	No	277 (87.1%)	582 (91.5%)	859 (90.1%)
	Yes	41 (12.9%)	54 (8.5%)	95 (9.9%)
Did things to scare or intimidate you purposely	No	270 (84.9%)	590 (92.8%)	860 (90.1%)
	Yes	48 (15.1)	46 (7.2%)	94 (9.9%)
Threatened to hurt you or someone you cared about	No	314 (98.7%)	629 (97.2)	948 (99.4%)
	Yes	4 (1.3%)	2 (0.3%)	6 (0.6%)
Sexual violence (*n* = 509)				
Physically forced you to have sexual intercourse	No	280 (88.1%)	605 (95.1%)	885 (92.8%)
	Yes	38 (11.9%)	31 (4.9%)	69 (7.2%)
Did you have sexual intercourse because you feared what he might do	No	252 (79.2%)	566 (89.0%)	818 (87.7%)
	Yes	66 (20.8%)	70 (11.0%)	136 (14.3%)
Did he force you to do something sexual that felt humiliating	No	266 (83.6%)	585 (92.0%)	851 (89.2%)
	Yes	52 (16.4%)	51 (8.0%)	103 (10.8%)

In addition, 129(40.6%) cases and 116(18.2%) of controls reported partner’s controlling behavior during their index pregnancy. Partner’s restriction of Health care (ANC) 75(23.6%), were the commonest form of partner’s controlling behavior reported (Table 4 show in a separate document).

**Table 4:** Partner’s controlling behavior among cases and controls who gave birth in public zonal hospitals of Tigray, Ethiopia, 2018 (*n* = 954)

Variables	Category	Cases *n* = 318 (%)	Control *n* = 636 (%)	Total *n* = 954(%)
Did he expect you to ask his permission before seeking Antenatal care for yourself	No	243 (76.4%)	556 (87.4%)	799 (83.8%)
	Yes	75 (23.6%)	80 (12.6%)	155 (16.2%)
Did he try to keep you from seeing your friends	No	306 (96.2)	617 (97.0%)	923 (96.8%)
	Yes	12 (3.8)	19 (3.0%)	31 (3.2%)
Did he try to restrict contact with your family of birth	No	308 (96.9%)	619 (97.5%)	927 (97.2%)
	Yes	10 (3.1%)	17 (2.7%)	27 (2.8%)
Did he ignore you and treats you indifferently	No	246 (77.4%)	578 (90.9%)	824 (86.4%)
	Yes	72 (22.6%)	58 (9.1%)	136 (13.6%)
Did he Insist on knowing where you are all time	No	250 (78.6%)	579 (91.0%)	829 (86.9%)
	Yes	68 (21.4%)	57 (9.0%)	125 (13.1%)
Did he get angry if you speak with another man	No	242 (76.1%)	572 (89.9%)	814 (85.3%)
	Yes	76 (23.9%)	64 (10.1%)	140 (14.7%)
Did he often get suspicious that you were unfaithful	No	289 (90.9%)	614 (96.5%)	903 (94.7%)
	Yes	29 (9.1%)	22 (3.5%)	51 (5.3%)

### The associations between intimate partner violence during pregnancy and adverse birth outcomes

As can be noted from the result of the bivariate analysis, all of the 5 variables showed a significant association with the adverse birth outcome at 5% level of significance. All of them were included for further analysis. Intimate partner violence, sexual violence, physical violence, psychological violence, and controlling behaviors were entered in the multivariable logistic regression at 0.05 level of significance.

A Multivariable logistic regression analysis was done by taking five variables in to account simultaneously to see significant association between intimate partner violence during pregnancy and adverse birth outcome. After an adjustment for age of women, marital status, occupation, area of residency, monthly income, educational status of women, ANC follow up, number of ANC visits, gravidity, and planned intention. The enter method regression was used after checking multicollinearity. Some of the variables which showed significant association with adverse birth outcomes in the bivariate analysis could not persist significantly in the multivariable analysis. These covariates were; psychological violence, sexual violence and controlling behavior.

In the multivariable binary logistic regression analysis, only two variables had shown a significant effect on the birth outcome at 5% level of significance.

Accordingly, it was found that the risk of PTB or LBW increased when the pregnant women were exposed to more than one type of violence. The odds of adverse birth outcome among those women had been exposed to more than one type of intimate partner violence during pregnancy were 3.12 times higher than those mothers who don’t experience more than one type of violence during pregnancy [AOR = 3.119; 95% CI (1.470, 6.618)].

Similarly, an association was also examined for subcategories of intimate partner violence (sexual, physical, and psychological) (Table 2). Physical violence during pregnancy was consistently associated with a significant increase in adverse birth outcome. It was observed that women who had been exposed to physical violence during pregnancy were 4.767 times at higher risk of having the adverse birth outcome as compared to those who had not [AOR = 4.767; 95% CI (2.515,9.034)]. (Table 5 show in a separate document).

**Table 5:** Bivariate and multivariable logistic regression analysis result for significant variables (*p* ≤ 0.05) in bivariate analysis in public zonal hospitals of Tigray, Ethiopia, 2017 (*n* = 954)

Variables	Category	Cases *n* = 318 (%)	Control *n* = 636 (%)	COR[95%CI]	AOR[95%CI]
Intimate Partner Violence	No	100 (31.4%)	465 (73.1%)	1	**1**
	Yes	218 (68.4%)	171 (26.9%)	5.928 [4.416,7.959] *	3.119 [1.470,6.618] *
Physical violence	No	183 (57.5%)	585 (92.0%)	1	**1**
	Yes	135 (42.5%)	51 (8.0%)	8.462 [5.890,12. 158] *	4.767 [2.515,9.034] *
Psychological violence	No	214 (67.3%)	520 (81.9%)	1	**1**
	Yes	104 (32.7%)	115 (18.1%)	2.197 [1.613,2.994] *	1.423 [0.732,2.764]
Sexual violence	No	248 (78.0%)	561 (88.9%)	1	1
	Yes	70 (22.0%)	75 (11.8%)	2.111 [1.475,3.021] *	0.798 [0.499,1.276]
Controlling behaviors	No	189 (59.4%)	520 (81.8%)	1	1
	Yes	129 (40.6%)	116 (18.2%)	3.060 [2.264,4.134] *	0.755[.479,1.191]

*P value <0.05 in bivariate analysis

On the other hand, in this study sexual violence, psychological violence and husband controlling behavior during pregnancy were not found to be associated with a significant increase in adverse birth outcome (Table 5 show in a separate document).

## Discussion

Intimate partner violence is an important public health, reproductive health, and social concern of the whole world. It could be a significant predictor of adverse outcomes for two individuals: The mother and her infant. Moreover, violence during pregnancy can have long-term consequences especially when it is under-recognized [15], This study was aimed to assess the association between intimate partner violence (IPV) during pregnancy and the adverse birth outcomes.

In this study, more than one-third of the women (40.8%) had been exposed to intimate partner violence during their index pregnancy period. The partners controlling behavior, was the most common violence with a magnitude of about 25.7% (95% CI 0.229, 0.285) followed by psychological violence 23.0% (95% CI 0.203, 0.257) physical violence 19.5% (95% CI 0.170, 0.220) and sexual violence 15.2% (95% CI 0.129, 0.175). This result is higher than the study results in Hosanna Town, Bale Zone, Moshi–Tanzania, South Carolina, Rwanda, Kenyan where 23, 25.8, 30.3, 35, 37% intimate partner violence were reported during index pregnancy period, respectively [18–21]. But, it is comparable with other systematic review of African studies where 2–57% of the mothers had been abused by their intimate partners during their pregnancy [16]. The variations in the reported IPV during pregnancy might be due to the differences in social beliefs on what constitutes IPV, tools and scales that were used for data collection and analyses and sample size and differences in study methodologies, parameters observed and the unwillingness of women to disclose.

The finding of this study also showed that women exposed to all type of intimate partner violence during pregnancy were three times more likely to experience LBW (AOR = 3.1; CI 95% [1.470,6.618]) and PTB (AOR = 2.5; CI 95% [2.198–2.957]) compared with women who don’t experience IPV during pregnancy. This result is congruent with the results from hospital-based case-control study in Southeast, Ethiopia LBW (AOR = 3.0 (95% CI; [1.57 to 7.18]) [21] and a prospective cohort study from Tanzania LBW (AOR = 3.2; CI 95%[1.3–7.7]) and preterm birth (AOR = 2.9; CI 95%[1.3–6.5]) [17] which found that partner violence against pregnant women increased low birth weight and preterm birth. Other studies have also exhibited consistent results [18], Vietnam [19], Iran [20], USA [10, 21, 22], USA [13, 16, 23].

This study also showed that physical violence during pregnancy was consistently associated with a significant increase in adverse birth outcome. It was observed that, women who had been exposed to physical violence during pregnancy were five times more likely to experience LBW (AOR = 4.767; CI 95% [2.515,9.034]) and PTB (AOR = 5.3; CI 95%: 3.95–7.094) as compared to those who were not exposed to physical violence. This is consistent with prior study conducted in Vietnam (Preterm birth (AOR = 5.5; 95%CI: 2.1–14.1) and LBW (AOR = 5.7; 95%CI: 2.2–14.9) [19],Tanzania PTB (AOR = 4.5; CI 95%: 1.5–13.7) and LBW (AOR = 4.8; CI 95%: 1.6–14.8) [17],USA [23, 24] demonstrating that experiencing physical violence during pregnancy is associated with a multitude of adverse pregnancy outcomes including LBW, PTB, and neonatal death. One possible reason is physical IPV may lead to traumas causing premature rupture of membranes or cause abruption of the placenta and subsequently PTB and LBW.

A slightly higher proportion of mothers of low birth weight and preterm infants reported having been abused sexually, psychologically and reported husband controlling behavior compared with controls, but the difference was not statistically significant. This agrees with the study conducted in Southeast Ethiopia which showed that sexually and psychologically are associated with adverse birth outcomes [25]. Perhaps one of the probable reasons of inconsistency with another study could be due to lack of a standard definition, regional variations in community awareness of violence during pregnancy and differences in study methodologies, setting, parameters observed and the unwillingness of women to disclose.

### Strength and limitation of the study

This study is based on a large sample of pregnant women. Thus, it has sufficient power to detect statistically significant differences between abused and non-abused women with and without adverse birth outcome. Besides poor birth outcomes, victims of IPV and their children may suffer long-term negative health effects. Therefore, this study could be very crucial to establish a standardized comprehensive intervention that promotes birth outcomes for victims of IPV.

Since the study was facility based and women who delivered at home were excluded in this study. Because of this reason, applicability of findings to the general population is uncertain. In addition, due to many ethical and social issues regarding physical and sexual violence, misreporting is conceivable. The sensitive nature of the topic and its proneness to response bias may lead to over- and under-reporting of the true extent of the abuse.

## Conclusion

The findings of this study revealed that overall intimate partner violence and physical violence during pregnancy was associated with a significant increase in low birth weight and preterm birth. But neither husband controlling behavior nor sexual and psychological violence exposure yielded significant associations with adverse birth outcome in this study.

Based on the study findings the following recommendations were forwarded to respective bodies. Health-care providers should be informed and aware of the possibility of violence as an underlying factor in women’s ill-health during pregnancy and should focus discussions on healthy relationships across the lifespan, with a particular focus on pregnant mother. Health extension workers should provide primary prevention in the form of prenatal education to inform couples about the risk of adverse birth outcomes in relation to IPV exposure. The regional Health bureau should train health care providers on how to screen, counsel, treat, and follow up of abused women. Efforts to address maternal and newborn health need to include issues of violence against women.

All health institution should Approach pregnancy care from a life course perspective necessitates that health care providers place greater emphasis on primary, preventive, pre-conceptional, and inter-conceptional care fundamental aspects of women’s overall health. Development of interventions to prevent violence against women from happening in the first place, including those that promote gender equality and safe and responsible relationships is also a critical need**.** National programs targeting violence against women should therefore emphasize that violence exercised during pregnancy has very serious health implications. The study further demands for longitudinal research regarding the pathway through which violence may lead to risk of adverse birth outcomes.

## Additional file

Additional file 1: Questionnaire for intimat partenr violence during pregnancy. (DOCX 25 kb)

## Abbreviations

AOR: Adjusted Odd Ratio
CI: Confidence interval
IPV: Intimate Partner Violence
IRB: Institution Review Board
SPSS: Statistical Package for Social Sciences
VAW: Violence against women
