# Supplementary material for: Intimate partner violence during pregnancy and adverse birth outcomes: a case-control study
Source: Reprod Health. 2019 Feb 25;16:22. doi: 10.1186/s12978-019-0670-4 (PMC6388467; doi:10.1186/s12978-019-0670-4)
Supplement: Supplementary file 1 — Questionnaire for intimat partenr violence during pregnancy. (DOCX 25 kb) [file 12978_2019_670_MOESM1_ESM.docx]

**Aksum University, College of Health Sciences, Department of Nursing**

Data collection tool for intimate partner violence (IPV) during pregnancy and adverse birth outcomes among women’s who gave birth in zonal hospitals of Tigray, Ethiopia

| **QUESTIONNAIRE FOR INTIMAT PARTENR VIOLENCE DURING PREGNANCY** | | | |
| --- | --- | --- | --- |
| **Name of Hospital** [__________________] | | | **Questionnaire Code**  [_______] |
|  | | **Questions and responses** | |
| **Q NO** | | **Section 1: Socio demographic characteristics** | |
| 101 | | How old are you? [_______] years | |
| 102 | | To which ethnic group do you belong?  [ 1] Tigray [ 2] Amhara [ 3] Afar [ 4] Other_________ | |
| 103 | | What is your current religion?  [ 1] Islam [ 2] Orthodox [ 3] Protestant [ 4] Catholic [ 5] others(specify_________) | |
| 104 | | Current marital status  [ 1] Never married [ 2] Married [ 3] Divorced [ 4] Separated [ 5] widowed | |
| 105 | | What is your educational level? (number of years in education)  [ 1] No education [ 3] Secondary (Grade 7-12)  [ 2] Primary school(Grade 1-6 ) [ 4] More than secondary (Abovegrade12) | |
| 106 | | Are you currently employed?  [ 1] Unemployed [ 2] Employed [ 3] Student [ 4] Retired | |
| 107 | | How much is your monthly income? [ _________ ETB] | |
| 108 | | Where do you live? [ 1] Urban [ 2] Rural | |
| **Pregnancy and obstetric characteristics pregnant women** | | | |
| 109 | How many pregnancies do you have? [_______] pregnancies | | |
| 110 | How many deliveries have you had?" [_______] deliveries | | |
| 111 | How many times do you have visited the ANC clinic for the current pregnancy? [_______] visits | | |
| 112 | Do you have a desire for this pregnancy?  [ 1] Yes [ 2] No | | |
| 113 | Does your partner have a desire for this pregnancy?  [ 1] Yes [ 2] No [ 8] I don’t know | | |

| **Section 2: psychological (emotional), physical and sexual violence**: | | | | | | | | | |
| --- | --- | --- | --- | --- | --- | --- | --- | --- | --- |
| When two persons married each other or live together they will share bad or good things. Even though they pass much of their lives, sometimes they may be in different mood, and may disagree with each other. This is common in many families. I would like to ask you some questions about this point. Some of the questions may be personal any way I would ask you to answer honestly. If someone interrupts us I will change our topic of discussion.  May I continue? ***If no thank the respondent and terminate the interview***  ***If Yes*** | | | | | | | | | |
| **Partners controlling behavior** | | | | | | | | | |
| 114 | | | I am now going to ask you some situations that are true for many. think about your (current or most recent) husband /partner would you say that it is generally true that he;   1. Try to keep you from seeing your friends [ 1] Yes [ 2] No 2. Tries to restrict contact with the family of birth [ 1] Yes [ 2] No 3. Insist on knowing where you are all time [ 1] Yes [ 2] No 4. Ignore you and treat you indifferently [ 1] Yes [ 2] No 5. Get angry if you speak with another man [ 1] Yes [ 2] No 6. Is often suspicious that you are unfaithful [ 1] Yes [ 2] No 7. Expect you to ask for permission before you seeking [ 1] Yes [ 2] No   (Antenatal care)health care for yourself | | | | | | |
| **Psychological (emotional) violence** | | | | | | | | | |
| Q no | Questions & filters | | | During current pregnancy | | | | | |
|  | **Has your current or most recent husband /partner ever….….** | | | 1=Never | 2=Sometimes, | 3=Usually | 4= Always | 7=refused/NR | |
| 115 | Insulted you or made you feel bad about yourself? | | | **[ 1 ]** | **[ 2 ]** | **[ 3 ]** | **[ 4 ]** | **[ 7 ]** | |
| 116 | Belittled or humiliated you in front of other people | | | **[ 1 ]** | **[ 2 ]** | **[ 3 ]** | **[ 4 ]** | **[ 7 ]** | |
| 117 | Done things to scare or intimidate you on purpose (e.g. the way he looked at you, by yelling ) | | | **[ 1 ]** | **[ 2 ]** | **[ 3 ]** | **[ 4 ]** | **[ 7 ]** | |
| 118 | Threatened when asking your friends/family) | | | **[ 1 ]** | **[ 2 ]** | **[ 3 ]** | **[ 4 ]** | **[ 7 ]** | |
| 119 | Threatened to hurt you or someone you care about | | | **[ 1 ]** | **[ 2 ]** | **[ 3 ]** | **[ 4 ]** | **[ 7 ]** | |
| **Physical violence** | | | | | | | | | |
| **Has your partner or any other person in the house ever….…** | | | | | | | | | |
| Q no | | Questions & filters | | During current pregnancy | | | | | |
|  | | **Has your current or most recent husband /partner ever….….** | | 1=Never | 2=Sths, | 3=Usually | 4= Always | 7=refused/NR | |
| 120 | | Slapped you or threw something at you that could hurt you? | | **[ 1 ]** | **[ 2 ]** | **[ 3 ]** | **[ 4 ]** | **[ 7 ]** | |
| 121 | | Push you or shoved you? | | **[ 1 ]** | **[ 2 ]** | **[ 3 ]** | **[ 4 ]** | **[ 7 ]** | |
| 122 | | Kicked, dragged you or beaten you up | | **[ 1 ]** | **[ 2 ]** | **[ 3 ]** | **[ 4 ]** | **[ 7 ]** | |
| 123 | | During pregnancy, hit your abdomen with a fist or with something else | | **[ 1 ]** | **[ 2 ]** | **[ 3 ]** | **[ 4 ]** | **[ 7 ]** | |
| 124 | | Strangled ,Choked or burnt you on purpose? | | **[ 1 ]** | **[ 2 ]** | **[ 3 ]** | **[ 4 ]** | **[ 7 ]** | |
| 125 | | Threatened to use or actually used a gun, knife or other weapon against you? | | **[ 1 ]** | **[ 2 ]** | **[ 3 ]** | **[ 4 ]** | **[ 7 ]** | |
| \| **Sexual violence:** \| \| --- \| | | | | | | | | | |
| \| **Has your partner or any other person in the house ever…** \| \| --- \| | | | | | | | | | |
| 126 | | Physically forced you to have sexual intercourse when you did not want to? | | **[ 1 ]** | **[ 2 ]** | **[ 3 ]** | **[ 4 ]** | | **[ 7 ]** |
| 127 | | Did you ever have sexual intercourse you did not want because you were afraid of what might be done to you? | | **[ 1 ]** | **[ 2 ]** | **[ 3 ]** | **[ 4 ]** | | **[ 7 ]** |
| 128 | | Did anyone ever forced you sexually in a way you did not approved | | **[ 1 ]** | **[ 2 ]** | **[ 3 ]** | **[ 4 ]** | | **[ 7 ]** |

**Section 3: check list for Adverse Birth outcomes**

| 129 | Birth weight | [_______ k.g ] |
| --- | --- | --- |
| 130 | GA | **[_______ wks]** |
| 131 | Apgar score in 1 Min | [_______] |
| 132 | Apgar score in 5 Min | [_______] |
| 133 | Neonatal problems | 1. Yes 2. No |
| 134 | Neonatal admission to ICU | 1. Yes 2. No |
| 135 | Neonatal death | 1. Yes 2. No |
